# Advantages and Limitations of Naturalistic Study Designs and Their Implementation in Alcohol Hangover Research

**DOI:** 10.3390/jcm8122160

**Published:** 2019-12-06

**Authors:** Joris C. Verster, Aurora J. A. E. van de Loo, Sally Adams, Ann-Kathrin Stock, Sarah Benson, Andrew Scholey, Chris Alford, Gillian Bruce

**Affiliations:** 1 Utrecht Institute for Pharmaceutical Sciences (UIPS), Faculty of Science, Division of Pharmacology, Utrecht University, 3584 CG Utrecht, The Netherlands; a.j.a.e.vandeloo@uu.nl; 2 Institute for Risk Assessment Sciences (IRAS), Faculty of Veterinary Medicine, Utrecht University, 3584 CM Utrecht, The Netherlands; 3 Centre for Human Psychopharmacology, Swinburne University, Melbourne VIC 3122, Australiaandrew@scholeylab.com (A.S.); 4 Addiction and Mental Health Group, Department of Psychology, University of Bath, Bath BA2 7AY, UK; sa221@bath.ac.uk; 5 Cognitive Neurophysiology, Department of Child and Adolescent Psychiatry, Faculty of Medicine, TU Dresden, 01307 Dresden, Germany; Ann-Kathrin.Stock@uniklinikum-dresden.de; 6 Psychological Sciences Research Group, University of the West of England, Bristol BS16 1QY, UK; chris.alford@uwe.ac.uk; 7 Education and Social Sciences, University of the West of Scotland, Paisley PA1 2BE, UK; Gillianbruce@gmail.com

**Keywords:** study design, naturalistic study, randomized controlled trial, alcohol, hangover, blinding, mobile technology

## Abstract

In alcohol hangover research, both naturalistic designs and randomized controlled trials (RCTs) are successfully employed to study the causes, consequences, and treatments of hangovers. Although increasingly applied in both social sciences and medical research, the suitability of naturalistic study designs remains a topic of debate. In both types of study design, screening participants and conducting assessments on-site (e.g., psychometric tests, questionnaires, and biomarker assessments) are usually equally rigorous and follow the same standard operating procedures. However, they differ in the levels of monitoring and restrictions imposed on behaviors of participants before the assessments are conducted (e.g., drinking behaviors resulting in the next day hangover). These behaviors are highly controlled in RCTs and uncontrolled in naturalistic studies. As a result, the largest difference between naturalistic studies and RCTs is their ecological validity, which is usually significantly lower for RCTs and (related to that) the degree of standardization of experimental intervention, which is usually significantly higher for RCTs. In this paper, we specifically discuss the application of naturalistic study designs and RCTs in hangover research. It is debated whether it is necessary to control certain behaviors that precede the hangover state when the aim of a study is to examine the effects of the hangover state itself. If the preceding factors and behaviors are not in the focus of the research question, a naturalistic study design should be preferred whenever one aims to better mimic or understand real-life situations in experimental/intervention studies. Furthermore, to improve the level of control in naturalistic studies, mobile technology can be applied to provide more continuous and objective real-time data, without investigators interfering with participant behaviors or the lab environment impacting on the subjective state. However, for other studies, it may be essential that certain behaviors are strictly controlled. It is, for example, vital that both test days are comparable in terms of consumed alcohol and achieved hangover severity levels when comparing the efficacy and safety of a hangover treatment with a placebo treatment day. This is best accomplished with the help of a highly controlled RCT design.

## 1. Introduction

The alcohol hangover is defined as a combination of mental and physical symptoms, experienced the day after a single episode of heavy drinking, starting when the blood alcohol concentration approaches 0 [1]. Studies in this research area examine the causes, functional consequences, and potential treatments of the next day (i.e., post-intoxication) effects of alcohol consumption. The alcohol hangover is associated with cognitive and psychomotor impairment [2] and mood changes [3], and may negatively affect daily activities, such as driving a car [4,5] or job performance [6]. The World Health Organization (WHO) estimates that 5.1% of the global burden of disease and injury is attributable to alcohol use and its consequences [7], and a recent UK study rated the economic costs of having hangovers in terms of absenteeism and presenteeism at 4 billion GBP per year [8]. Despite this, the pathology of the alcohol hangover is poorly understood [9,10], and although there is great market demand [11], there are currently no effective hangover treatments available [12].

Both randomized controlled trials (RCTs) and naturalistic study designs are commonly applied in hangover research. Although increasingly applied in social sciences and medical research, the suitability of using naturalistic study designs remains a topic of debate. To examine this, our paper compares the naturalistic study design with the traditional controlled experimental design, in particular RCTs. It discusses the advantages and disadvantages of both designs and suggests solutions for issues of concern.

Traditionally, medical science has been based on clinical observations of patients and control samples. In the fields of psychiatry and psychology, for example, participants either self-report their mood or an investigator observes their behavior. This was common practice before the introduction of RCTs. However, since their introduction, the quality, methodology, and reporting of medical science has been continuously optimized [13], and the RCT is, therefore, currently often viewed as the gold standard that allows for the most precise and systematic investigations. RCTs are, for example, commonly used to investigate the efficacy and safety of a medicinal drug in a specific patient population. The RCT design is characterized by having several inclusions, exclusion, and discontinuation criteria that apply to participants, including lifestyle rules with regard to, for example, alcohol and drug use and smoking. RCTs are ideally double or triple blind to avoid influencing the study outcome, and participants are randomly allocated to treatment conditions. The treatment order is varied (cross-over) to account for any learning or order effects. All study-related activities are highly standardized and conducted per protocol, with the aim to have all test days as identical to each other as possible. In theory, the only methodological difference between the test days is the administered treatment or intervention. This way, it is thought that the study gathers ‘clean’ data about the effect of the treatment or intervention. However, this level of control comes at the cost of RCTs creating highly artificial situations, which lack ecological validity and/or potentially differ from the effects observed in the participants’ everyday life.

On the other hand, the aim of the naturalistic study design is to mimic real-life as closely as possible, and as such is characterized by a minimum of lifestyle rules for participants, in which the investigators do not (actively) interfere with their activities. Hence, several behaviors and activities of the participants are not standardized and not regulated by a study protocol. Participants continue their normal lives and may visit the testing site for assessments or bio-sample collection or may even be able to undertake these assessments whilst remaining in their usual environment. Commonly, the only instruction is to behave normally (e.g., take their medication as prescribed or drink alcohol as they would on a normal night out), complete scheduled assessments (e.g., a sleep diary or online scales), and visit the testing site at set times.

The naturalistic design is increasingly utilized in various research areas and has been successfully applied in phase III studies and pharmacovigilance research, e.g., to investigate the efficacy of antipsychotics in schizophrenia patients [14] or breast cancer patients [15]. The following sections will discuss the commonalities and differences between RCTs and naturalistic study designs, advantages and disadvantages, and possible solutions to common pitfalls.

## 2. Recruitment, Screening, and Test Days

Both RCTs and naturalistic studies have highly controlled data collection on test days. This includes conducting standardized and validated tests according to good clinical practice (GCP) and utilizing standard operating procedures at pre-set times specified in the study protocol. Furthermore, both study designs can have various lifestyle rules (e.g., no alcohol or drug use, no smoking), which can be verified by objective assessments on the test day. In this respect, naturalistic studies do usually not differ from RCTs.

Recruitment, screening, selecting, and training of participants can also be equally rigorous in RCTs and naturalistic studies. Both study designs can apply the same inclusion and exclusion criteria. Objective assessments can be conducted to verify the criteria (e.g., blood chemistry, urinalysis, and electrocardiography), and participants can be familiarized with and trained in completing psychometric tests, treatment administration, and completing mood scales. The main reason that rigorous screening and selection of study participants in RCTs is common is that it ensures a more homogenous study sample. It is expected that there will be more variability between study participants in responsiveness to the administered treatments when the eligibility criteria are loosened. Loosening eligibility criteria may then decrease the chances of successfully demonstrating efficacy or safety. To demonstrate the true drug effect, assessments should not be obscured by various external uncontrolled factors. Unfortunately, applying a large number of eligibility criteria usually results in a considerable number of screening failures (i.e., participants not meeting all criteria for participation) or drop-outs and compliance failures (i.e., participants discontinuing or failing to adhere to the study protocol). This is commonly seen in RCTs [16,17,18]. In addition, a number of people may not participate in the first place when they are informed about the strict lifestyle rules and the hassle of screening procedures (e.g., blood drawings and medical examinations). Unfortunately, this may induce a (self-)selection bias in the study sample.

The extent to which RCT participants in drug development are representative for the patient population can therefore be questioned [17,18]. While some ‘safety-related’ eligibility criteria are obviously necessary, other eligibility criteria (e.g., cut off values for body weight ranges) are often not strongly justified by supporting scientific evidence [16]. Not applying or loosening unjustified eligibility criteria will increase recruitment speed and result in a study sample that better reflects the entire patient population. Some recent RCTs have, therefore, included a ‘real life’ arm in their study, including participants who did not meet the stringent eligibility criteria of the RCT [19]. As naturalistic studies aim to mimic real life, eligibility criteria are often less strict than those applied in RCTs. This may significantly increase the ecological validity of the study, which is usually low in RCTs [14].

## 3. Level of Control, Supervision, and Monitoring

All RCT study-related activities are closely monitored at the testing site (e.g., clinic or lab). However, this is not always the case in naturalistic study designs, in which researchers are not necessarily present.

One issue is not reporting behavior. As participation in research studies is typically confidential, and sometimes anonymous, there should be no objective reason for participants not to report certain behaviors. However, if these behaviors are restricted by discontinuation criteria, participants may decide not to report them in order to prevent themselves from being excluded from further study participation. Another reason could be social desirability, as participants may be less likely to report behaviors or incidents that they either perceived to be detrimental to their self-image or that they fear may result in negative judgement from others. Another issue may be misreporting. Participants may not report certain behaviors simply because they were not asked about them (e.g., a researcher refrains from questioning participants about drug use, because an inclusion criterion to participate in the study was not using drugs), or they view these behaviors as irrelevant to the study (e.g., a participant being unaware that drinking a cup of coffee can improve subsequent cognitive test performance). Fortunately, there are several ways to retrospectively and objectively verify the occurrence of study-relevant behaviors, including assessments for residual alcohol use (breathalyzer), drug use (urine tests), and recent smoking (exhaled carbon monoxide), or monitor activity and sleep episodes (actigraphy).

In both naturalistic studies and RCTs, it is also increasingly common to implement ambulatory assessments in the study design, for example cognitive tests or questionnaires completed online/at home. The advantage of not having to schedule visits to the testing site makes it easier to participate in the study and thus reduces chances of dropouts. It also allows for repeated testing at fixed time intervals, which may help to reduce the risk of study-relevant events not being recalled correctly. At home, testing has been successfully implemented in numerous phase III studies, using the same tests that would have been conducted in the clinic (e.g., online cognitive tests, blood pressure assessment, or self-administered blood glucose tests). In short, the use of mobile technologies enable compliance monitoring. Furthermore, mobile technology, home testing, and the internet provide various ways to ensure valid and reliable real-time assessments of cognitive and physical functioning, mood, and biomarkers [20,21,22,23].

However, in naturalistic studies, assessments are often limited to retrospective and subjective self-reports. When relying entirely on self-reports, recall bias and memory loss may have a significant impact on the accuracy of the collected data. For example, research has shown that people under- or over-estimate the amount of alcohol consumed [22,23] and that subjective and objective assessment of sleep parameters are not always in concordance with each other [23]. The latter should be taken into account when interpreting the data obtained in naturalistic studies.

To prevent the presence of observers/researchers from influencing the behaviors of study participants, one could consider monitoring the subject’s behaviors in real time via video streaming, without the awareness of study participants that they are being filmed. However, this approach would raise ethical, privacy, and data security concerns. A better alternative to this would be to apply mobile technology to objectively measure behaviors, including parallel objective measures to help triangulate data obtained from other measures.

Activity, sleep, and physiological parameters, such as heartrate and body temperature, can, for example, all be measured in real time using activity watches or ‘wearables’. Behavioral and mood data can be collected by real-time self-reports via smartphone apps (e.g., entering every drink they consumed). Alternatively, wearable technology (watches) that may record transdermal alcohol concentrations are currently being developed. In the future, these devices could be used to complement or partly replace self-reports. Moreover, they could help to reduce drop-out rates as a number of “passive” measurements could be conducted without requiring any effort from the participants. Importantly, this would also help to obtain a more complete picture in studies that investigate aversive effects, such as a hangover, which might lead to systematic drop-outs on the more severe end of the symptom scale. Taken together, mobile technology would not only reduce the strain on study participants, but potentially also make the measurements more objective. In addition, test batteries used in RCTs are often administered as single assessments or, at best infrequently. These can therefore easily miss critical events or periods. Mobile data collection can include participant actioned recording of events and more regular testing, or continuous psychophysiological assessments, including wearable devices, which can all provide a better picture of participant behavior and subjective state.

As part of mobile testing, conducting an online survey is another common way to collect data from participants. This is effective if the subject sample is large or if it is not necessary or possible for participants to visit the research facility (e.g., due to obstacles, such as bad weather, large distances, or physiological constraints). While online methodologies are an easy way to collect data, there are several disadvantages. For example, the researcher cannot be certain whether the scheduled participant is completing the survey or whether someone else is doing it in their place. Furthermore, the condition of the participant cannot be verified by the researcher (e.g., they might be drunk or drugged while completing the survey or may not be giving the assessment their full attention), which may reduce the accuracy and validity of the resulting data. Further enhancing this methodology can increase reliability of the collected data, for example by video streaming. Video streaming can confirm if the scheduled participant is actually present and can verify how the participant conducts a test or completes questionnaires. It further enables the researcher to observe the general health and makes it possible to record real-time observer-rated adverse effects.

## 4. Level of Standardization of Tests and Procedures

While the scrutiny of recruitment, screening, and test day assessments can have comparable levels of control and standardization in RCTs and naturalistic studies, the designs differ significantly with regard to the standardization and activities of participants during the intervention phase. In RCTs, every activity of the participant takes place in the testing facility. Activities are scheduled at pre-set times and conducted according to standard procedures. This includes treatment administration, meals, activities, time going to bed, or the environment where participants spend time (i.e., the testing site). Moreover, all assessments and activities are standardized and precisely monitored and recorded by the researchers. The rationale to conduct an RCT in this way is clear: By minimizing the non-intervention-related variability (i.e., the uncontrolled “noise”) in all potentially study-relevant parameters, the chance of observing a true treatment effect increases.

In contrast, in naturalistic studies, participants continue with their usual activities and researchers do not observe them or provide instructions on how to behave. Thus, the researchers do not interfere with the participants’ activities. Consequently, behaviors are unstandardized and self-initiated. The rationale for this approach is to closely mimic real life, i.e., to maximize ecological validity. This ecological validity is important because it best reflects the way in which phenomena, such as hangovers, emerge, and medicinal treatments will be actually used when marketed. Additionally, eligibility criteria in naturalistic studies may be less strict compared to those of RCTs to ensure the study sample better reflects the heterogeneous population who will use a treatment or intervention in clinical practice and provide a better picture of efficacy. Thus, rather than a limitation, the lack of standardization can be considered to be a benefit of the naturalistic study design.

A related discussion is the use of subjective versus objective assessments and the quest for the inclusion of biomarker assessments in a study. Cytokine concentrations, for example, can vary in cases of depression [24] or during the hangover state [25]. It can thus be interesting to assess cytokine changes in blood or saliva. The alcohol hangover state is a subjective experience which, up till now, cannot be objectively measured. Although this can be viewed as a significant limitation of this research area, it should be underlined that biomarkers are per definition (at best) proxy-measures if one aims to measure mood or how the participant feels. Clinical observations may be an alternative, but these usually do not substitute for subjective assessments of the severity or nature of mood states. To date, the best way to rate mood levels is by asking participants to report how they feel [26]. Interestingly, in this regard, the outcome of these subjective assessments is not always in correspondence with the outcome of objective biomarker assessments. Participants can, for example, report feeling perfectly fine while having a clinically relevant increase in blood pressure. Alternatively, participants can report sleep complaints and poor sleep quality while their polysomnographic outcomes are within normal ranges. Together, these findings advocate to include both subjective and objective assessments in future studies, irrespective of whether the study design is RCT or naturalistic.

## 5. Implications for Hangover Research

To provoke the hangover state, an evening of supervised alcohol consumption is typically scheduled in RCTs. The amount and type of alcoholic drink (and placebo) and the pace of drinking are usually pre-defined, and drinking is conducted within a pre-set time frame. This is typically conducted in a clinical setting, often accompanied by other participants who do not know each other. Food and other beverage intake (e.g., water) are prohibited or controlled, as are the cognitive and physical activities of the participants. All activities are closely monitored and recorded by the researchers, including blood alcohol concentration (BAC) assessments to verify alcohol consumption levels and adverse event recording. The evening activities are often concluded by a night of supervised sleep in the clinic, with a pre-set bed-time and wake-up time. Sleep quality and duration can be monitored with polysomnography or study personnel.

In contrast, in naturalistic studies, participants drink in a familiar setting (e.g., a bar or at home) with people they know, engaging in their usual activities. These normally differ from activities employed in RCTs (e.g., dancing in a club versus reading a magazine in the laboratory). In naturalistic studies, participants can eat food when they feel hungry and smoke and are exposed to external stimuli which are not replicated in the RCT setting (e.g., visiting multiple bars, walking outside in the rain, waiting for a bus to travel home). They can go to bed when feeling sleepy without being restricted by study procedures, which often dictate a much earlier time-to-bed than people have in real life after an evening out. As they sleep in their own beds, they will not experience the sleep problems that are common in RCTs, in which participants sleep in a new and unknown clinical environment (e.g., the first night effect) [27,28]. In addition, participants can apply their personal sleep habits, sleep hygiene activities, and wake-up rituals in naturalistic studies. Finally, socializing, expectancies, and motives for alcohol consumption most likely differ between real-life situations and RCTs and may impact assessment outcomes. Thus, in naturalistic studies, participants can either drink alone or have an evening with friends in a setting of their own choice. Bedtime is self-initiated, and participants sleep at home in their own bed. The next morning, participants come to the testing site for the assessments on the test day. Past evening behaviors are recorded retrospectively (e.g., via questionnaires or an interview), and in case of mobile technology use, objective data read-outs are obtained from the devices.

Whether or not it is important to monitor the drinking session depends entirely on the aim of individual research projects. For some studies, it may be essential that certain behaviors are strictly controlled. For example, when comparing the efficacy and safety of a hangover treatment with a placebo treatment, it is vital that both test days are comparable, in terms of consumed alcohol and achieved hangover severity levels. In this case, a strictly controlled RCT design would be favorable. If one chooses to use a naturalistic study design in efficacy studies, a statistical analysis should account for differences between the test days (e.g., in the form of co-variates or propensity scores). However, it is not always possible to accurately account for all variables. This could, for example, be because they depend on subjective self-reports (e.g., alcohol intake), because certain information is lacking (e.g., congener content of drinks), or because a certain factor has not (yet) been recognized as relevant (e.g., a certain genotype or developmental factors). In summary, several important factors that differ between test days (e.g., certain behaviors) that may bias the comparison between treatment and placebo will likely remain unknown or unrecognized and, therefore, not properly accounted for.

On the other hand, if one is primarily interested in the effects of the (subjective) alcohol hangover itself on cognitive performance, mood, or other variables, then the behaviors that provoked the hangover state are of limited importance. In this case, there is no clear need to monitor the amount and type of alcohol consumed, estimated peak BAC, and the setting and behaviors during the drinking session. In extremis, participants could then be recruited in the morning after an evening out and allocated to a hangover or control group, or groups that consumed alcohol or not. This would be the ultimate way of not interfering with participant drinking behavior, as participants were unaware that they were going to participate in a research study at the time they displayed the study-relevant behavior (e.g., drinking or staying sober). This design was successfully applied by Devenney et al. [29], who recruited participants at university venues in the morning, i.e., on the day following the drinking session. However, if one is interested in how drinking variables and behaviors during the drinking session cause or relate to hangover variables, it is essential that these are accurately measured. Statistical analysis can then take into account the observed interindividual differences in naturalistic studies.

There are obvious advantages of applying a naturalistic study design in alcohol hangover research, as the drinking session reflects what people do in normal life. In contrast to RCTs, they are not forced to adapt to a drinking regime, including consuming alcoholic beverages that are not their regular choice during a pre-set drinking time period that may differ from a normal night out. In fact, research consistently shows that in real life situations, most people consume much larger quantities of alcohol over a longer period of time, as compared to the pre-set dosages of alcohol that are administered in clinical studies to provoke a hangover. This results in significantly higher (and more realistic) BAC levels in naturalistic studies, as compared to many RCTs [30].

Assessments during the hangover state can then take place in the clinic, following a highly standardized and controlled protocol, similar to RCTs. Alternatively, Scholey et al. [31] utilized online cognitive testing in a naturalistic hangover study and demonstrated that this was an effective way to collect objective data in real time during the hangover state. This study also addressed the issue of participant drop out. It has been argued that participants who experience severe events may not continue participation in naturalistic studies. This would of course bias the study outcome in favor of a treatment. Scholey et al. [31] compared their study participants with their dropouts. For both groups, peak BAC was assessed in real time the evening before the (hangover) test day, and no significant BAC difference was observed between participants who did and did not complete the test day assessments. Hence, there are presumably other reasons than mere degree of intoxication that determine whether participants discontinue study participation or not. A different approach has been the use of mobile technology, including screen-based tests, to enable participants to be assessed within the privacy and safety of their own homes, without the need to travel to the test center when hungover, avoiding dropouts [32].

Finally, studies comprising alcohol administration to humans usually require ethics approval. For many ethics committees, it appears that a noteworthy difference is made based on whether the alcohol is actually administered to participants by the experimenters (RCTs) or whether they administer it themselves in an unsupervised setting (naturalistic studies). Ethics committees often limit the amount of alcohol researchers are allowed to administer to participants of RCTs to a blood alcohol concentration (BAC) below 0.12%, while in study protocols for naturalistic studies it is unknown how much alcohol participants will consume. Naturalistic studies consistently demonstrate that actual drinking levels are associated with much higher BACs. For example, Hogewoning et al. [30] reported an estimated BAC of 0.2%. When interviewing naturalistic study participants, they attest that they had a ‘normal’ night out, including their usual drinking behavior. This is an odd situation considering that, in RCTs, alcohol consumption is closely monitored with a physician and study personnel present, while participants can drink alcohol freely and unsupervised in naturalistic studies. Monitoring the level of alcohol consumed will also aid in evaluating hangover treatments. True symptom levels may not be assessed in the laboratory due to alcohol dosing restrictions, where effectively only ‘sub-clinical’ hangover symptom levels are evaluated.

Of note, viewpoints and safety concerns of ethics committee members are not always in agreement with those of study participants. For example, Petrie et al. [33] investigated the stress and pressure/imposition experienced by RCT participants for a variety of study-related handlings (e.g., blood pressure assessment, blood drawing) and compared their ratings to those of ethics committee members. The study revealed that several commonly applied procedures, such as taking a saliva sample or completing a questionnaire or mood scale, were rated as significantly less stressful by RCT participants compared to the ratings anticipated by ethics board members.

Petrie et al. [33] also compared the experienced stress levels in RCT procedures with those experienced in daily life and found that many relatively harmless experiences (e.g., stress when ‘asked to donate to a charity in the street’ or being ‘caught in the rain’) were rated as more stressful by study participants than completing a mood scale or delivering a saliva or urine sample. The overall conclusion of the study was that study-related stress and the impact of procedures in the standardized data collection may be overestimated by some ethics committees. Unfortunately, the restrictions that ethics committees feel inclined to impose upon proposed research projects (especially RCTs) can have a significant impact on the ecological validity of these studies and the consequential validity of the findings.

## 6. Concluding Remarks

The commonalities and differences between RCT designs and naturalistic studies are summarized in Table 1.

RCT designs are preferred for studies that require strictly-controlled study procedures. Treatment efficacy and safety studies, for example, require controlled treatment administration and the variability in participants’ behaviors (e.g., alcohol intake, physical activity, food intake, and sleep) should be kept to a minimum. However, RCTs, per definition, modify and structure participant behaviors in a standardized and, therefore, often “unnatural” way. Therefore, a naturalistic study design is preferred if one aims to better understand or mimic real-life interventions. The lack of standardization of naturalistic studies should, therefore, be considered as a benefit of the study design.

Additionally, free drinking in naturalistic studies often exceeds the intoxication limits deemed safe and ethically approved for RCT studies, which further increases the ecological validity of naturalistic hangover studies compared to RCT hangover studies. To improve the level of control in naturalistic studies, mobile technology can be used to assess objective real-time data and control the quality of assessment, without investigators interfering with participant behaviors.

## Figures and Tables

**Table 1:** Commonalities and differences between randomized controlled trial (RCT) and naturalistic study designs.

**Study Design**	**RCT**	**Naturalistic Study Design**
Ecological validity	Low to medium	High
External validity	Low to medium	High
Internal validity	High	Low to medium
Criterion validity	High	High
Construct validity	High	High
**Screening**	**Controlled, per protocol**	**Controlled, per protocol**
Inclusion, exclusion criteria	Yes	Yes
Familiarize with procedures and tests	Yes	Yes
**Drinking session**	**Per protocol, supervised**	**Uncontrolled, not supervised**
Instructions	Per protocol	None, or minimal
Amount, type of drink	Pre-set, standardized	Self-initiated
Start and stop time of drinking	Pre-set, per protocol	Self-initiated
Behaviors during drinking	Restricted, per protocol	Free (as normal)
Drinking environment	Research unit	Free (e.g., bar(s), at home)
Social aspects of drinking (if not alone)	With strangers	With friends or strangers
Real time assessments (e.g., BAC)	Yes	Possible via mobile technology
Food and water intake	Restricted, per protocol	Free (as normal)
Smoking	Restricted, per protocol	Free (as normal)
**Sleep**	**Controlled and supervised**	**Uncontrolled, not supervised**
Time to bed, wake up time	Restricted, per protocol	Self-initiated
Sleep hygiene and related behaviors	Restricted, per protocol	Self-initiated
Monitoring, real time assessments	Yes	Possible via mobile technology
Sleep environment	Sleep unit	At home or elsewhere
**Test days**	**Controlled and standardized**	**Controlled and standardized**
Psychometric tests, mood assessments	Standardized and validated	Standardized and validated
Time of testing	Per protocol at pre-set time	Per protocol at pre-set time
Conductance of study procedures	Per protocol	Per protocol
Supervision, monitoring	Yes	Yes

Description of validity types: Ecological validity = to what extend the study reflects a realistic hangover drinking occasion; external validity = to what extent can findings be generalized to the population as a whole; internal validity = to what extent can the design demonstrate causal effects; criterion validity = to what extent are measures related to study outcomes; construct validity = the degree to which the administered tests measure what they claim or purport to be measuring. Abbreviation: Blood alcohol concentration (BAC). Please note that this table is intended to contrast the RCT and naturalistic study design. Some studies might incorporate features of both designs (e.g., supervised and standardized alcohol administration, but unsupervised sleep at home). Additionally, studies with the same design type may differ significantly in the levels of control, standardization, and quality.

## References

[1] Van Schrojenstein Lantman M., van de Loo A.J.A.E., Mackus M., Verster J.C. (2016). Development of a definition for the alcohol hangover: Consumer descriptions and expert consensus. Curr. Drug Abuse Rev..

[2] Gunn C., Mackus M., Griffin C., Munafò M.R., Adams S. (2018). A systematic review of the next-day effects of heavy alcohol consumption on cognitive performance. Addiction.

[3] McKinney A. (2010). A review of the next day effects of alcohol on subjective mood ratings. Curr. Drug Abuse Rev..

[4] Verster J.C. (2007). Alcohol hangover effects on driving and flying. Int. J. Disabil. Hum. Dev..

[5] Verster J.C., Bervoets A.C., de Klerk S., Vreman R.A., Olivier B., Roth T., Brookhuis K.A. (2014). Effects of alcohol hangover on simulated highway driving performance. Psychopharmacology.

[6] Frone M.R., Verster J.C. (2013). Alcohol hangover and the workplace: A need for research. Curr. Drug Abuse Rev..

[7] World Health Organization (2018). Global Status Report on Alcohol and Health 2018.

[8] Bhattacharya A. (2019). Financial Headache. The Cost of Workplace Hangovers and Intoxication to the UK Economy.

[9] Penning R., van Nuland M., Fliervoet L.A., Olivier B., Verster J.C. (2010). The pathology of alcohol hangover. Curr. Drug Abuse Rev..

[10] Tipple C.T., Benson S., Scholey A. (2016). A Review of the Physiological Factors Associated with Alcohol Hangover. Curr. Drug Abuse Rev..

[11] Mackus M., Van De Loo A.J., Nutt D., Verster J.C., Lantman M.V.S. (2017). An effective hangover treatment: Friend or foe?. Drug Sci. Policy Law.

[12] Jayawardena R., Thejani T., Ranasinghe P., Fernando D., Verster J.C. (2017). Interventions for treatment and/or prevention of alcohol hangover: Systematic review. Hum. Psychopharmacol. Clin. Exp..

[13] Brunoni A.R., Tadini L., Fregni F. (2010). Changes in clinical trials methodology over time: A systematic review of six decades of research in psychopharmacology. PLoS ONE.

[14] Fagiolini A., Rocca P., De Giorgi S., Spina E., Amodeo G., Amore M. (2017). Clinical trial methodology to assess the efficacy/effectiveness of long-acting antipsychotics: Randomized controlled trials vs naturalistic studies. Psychiatry Res..

[15] Fleming L., Randell K., Stewart E., Espie C.A., Morrison D.S., Lawless C., Paul J. (2019). Insomnia in breast cancer: A prospective observational study. Sleep.

[16] Van Spall S.H.G., Toren A., Kiss A. (2007). Eligibility criteria of randomized controlled trials published in high-impact general medical journals. JAMA.

[17] Roehrs T., Verster J.C., Koshorek G., Withrow D., Tancer M., Roth T. (2018). How representative are insomnia clinical trials?. Sleep Med..

[18] Huls H., Abdulahad S., van de Loo A.J.A.E., Mackus M., Roehrs T., Roth T., Verster J.C. (2018). Inclusion and exclusion criteria of clinical trials for insomnia. J. Clin. Med..

[19] Björkholm C., Gannedahl A., Cars T., Rive B., Merclin N., Umuhire D., Milz R., van Sanden S., Lööv S.-A., Hellner C. (2020). Constructing a real-world evidence arm to quantify the placebo effect in a randomized control trial utilizing electronic-medical record depression scales. Eur. Neuropsychopharmacol..

[20] Verster J.C., Tiplady B., McKinney A. (2012). Mobile technology and naturalistic study designs in addiction research. Curr. Drug Abuse Rev..

[21] Adams Z., McClure E.A., Gray K.M., Danielson C.K., Treiber F.A., Ruggiero K.J. (2017). Mobile devices for the remote acquisition of physiological and behavioral biomarkers in psychiatric clinical research. J. Psychiatr. Res..

[22] Monk R.L., Heim D., Qureshi A., Price A. (2015). “I have no clue what I drunk last night” using Smartphone technology to compare in-vivo and retrospective self-reports of alcohol consumption. PLoS ONE.

[23] Devenney L.E., Coyle K.B., Roth T., Verster J.C. (2019). Sleep after heavy alcohol consumption and physical activity levels during alcohol hangover. J. Clin. Med..

[24] Janssen D.G.A., Verster J.C., Baune B.T. (2010). Depression, immunity and cytokine function: A psychoneuroimmunological review on depression and pharmacological response. Hum. Psychopharmacol..

[25] Van de Loo A.J.A.E., Mackus M., Knipping K., Brookhuis K.A., Kraneveld A.D., Garssen J., Verster J.C. (2017). Changes over time in saliva cytokine concentrations the day following heavy alcohol consumption. Allergy.

[26] Baldwin W. (1999). Information no one else knows: The value of self-report. The Science of Self-Report.

[27] Webb W.B., Campbell S.S. (1979). The first night effect revisited with age as variable. Waking Sleep..

[28] Le Bon O., Staner L., Hoffmann G., Dramaix M., San Sebastian I., Murphy J.R., Kentos M., Pelc I., Linkowski P. (2001). The first-night effect may last more than one night. J. Psychiatr. Res..

[29] Devenney L.E., Coyle K.B., Verster J.C. (2019). Memory and attention during an alcohol hangover. Hum. Psychopharmacol..

[30] Hogewoning A., van de Loo A.J.A.E., Mackus M., Raasveld S.J., De Zeeuw R., Bosma E.R., Bouwmeester N.H., Brookhuis K.A., Garssen J., Verster J.C. (2016). Characteristics of social drinkers with and without a hangover after heavy alcohol consumption. Subst. Abuse Rehabil..

[31] Scholey A., Benson S., Kaufman J., Terpstra C., Ayre E., Verster J.C., Allen C., Devilly G. (2019). Effects of alcohol hangover on cognitive performance: A field/internet mixed methodology approach. J. Clin. Med..

[32] Alford C., Martinkova Z., Johnson S., Tiplady B., Verster J.C. (2015). The effects of alcohol hangover on performance and mood including risk taking when assessed at home. Alcohol. Clin. Exp. Res..

[33] Petrie K.J., Faasse K., Notman T.A., O’Carroll R. (2013). How distressing is it to participate in medical research? A calibration study using an everyday events questionnaire. J. R. Soc. Med. Short Rep..

